# Operating Table Height Optimization Reduces Surgeon Postural Load During Total Knee Arthroplasty: An Ergonomic Simulation Study

**DOI:** 10.3390/jcm15072782

**Published:** 2026-04-07

**Authors:** Marina Sánchez-Robles, Carmelo Marín-Martínez, Vicente J. León-Muñoz, Joaquín Moya-Angeler, Francisco Lajara-Marco

**Affiliations:** 1 Department of Orthopaedic Surgery and Traumatology, Hospital General Universitario Rafael Méndez, Lorca, 30817 Murcia, Spain; marina24sr@gmail.com; 2 Department of Orthopaedic Surgery and Traumatology, Hospital General Universitario Reina Sofía, 30003 Murcia, Spainvleonmd@gmail.com (V.J.L.-M.); drlajaramarco@gmail.com (F.L.-M.); 3 Instituto Murciano de Investigación Biosanitaria Pascual Parrilla (IMIB), 30120 Murcia, Spain; 4 Instituto de Cirugía Avanzada de la Rodilla (ICAR), 30005 Murcia, Spain; 5 Olympia Medical Centre, 28046 Madrid, Spain

**Keywords:** ergonomics, occupational health, musculoskeletal disorders, orthopaedic surgery, total knee arthroplasty, Rapid Entire Body Assessment

## Abstract

**Background**: Work-related musculoskeletal disorders (WMSDs) are prevalent among orthopaedic surgeons as a result of prolonged exposure to non-neutral postures and forceful manual tasks during surgery. Although working height is a key determinant of trunk and upper-limb posture, the systematic evaluation of ergonomic working-height recommendations in orthopaedic surgery remains limited. **Methods**: A simulated left total knee arthroplasty (TKA) was divided into twelve critical surgical steps and analysed across four commonly used surgeon positions (A–D). Two conditions were compared: uncorrected working height (N) and working height corrected according to Canadian Centre for Occupational Health and Safety (CCOHS) recommendations (C). Joint angles were measured from standardized photographs using Kinovea software, and postural load was quantified with the Rapid Entire Body Assessment (REBA) method. Two trained evaluators conducted three independent assessments, yielding 288 REBA scores. **Results**: Mean REBA scores decreased across all surgeon positions following ergonomic correction, with statistically significant reductions observed in positions A, B, and D. When pooled across all position–step combinations (n = 48), the mean reduction was 0.92 REBA points (95% CI 0.50–1.33; *p* < 0.001). Notably, 27 of the 48 position–step comparisons exceeded the minimal detectable change threshold. The largest reductions occurred during force-intensive surgical steps, including bone cutting, drilling, and implant impaction. **Conclusions**: Adjusting working height in accordance with CCOHS ergonomic recommendations reduces surgeons’ postural load during TKA. These findings support the integration of evidence-based ergonomic adjustments into routine orthopaedic surgical practice.

## 1. Introduction

Work-related musculoskeletal disorders (WMSDs) are common among operating theatre professionals due to prolonged exposure to awkward postures, repetitive movements, and physically demanding tasks. The literature indicates that more than 60% of surgeons develop some form of WMSD during their career, with predominant involvement of the cervical spine, lumbar region, and shoulders [1,2]. Orthopaedic surgeons are at particularly high risk, as many procedures require the manipulation of heavy instruments and the maintenance of extreme postures for prolonged periods. Several studies have reported a high prevalence of musculoskeletal pain in this group, together with limited training and insufficient implementation of ergonomic measures [3,4]. Although general recommendations aimed to improve operating theatre ergonomics exist, specific evidence in orthopaedic surgery remains limited. Recent frameworks and institutional initiatives have underscored the importance of enhancing occupational health and ergonomic practices in surgical environments. These efforts highlight the need for systematic assessment and preventive strategies in orthopaedic surgery and other high-demand specialties. For example, the Ergonomic Risk Assessment Model (ERAM) has been proposed as a framework to promote fair healthcare practices and improve working conditions in orthopaedic settings [5].

In recent years, simple interventions have been proposed, such as modifying instrument layout, optimising workspace organisation, or adjusting the working surface height, to reduce the physical burden on the surgeon [6,7,8,9,10]. However, these measures have rarely been assessed quantitatively. Working height is a well-recognised factor that directly influences trunk and upper-limb posture. Although the Canadian Centre for Occupational Health and Safety (CCOHS) provides recommended working heights for task types, their application in the surgical setting has not been evaluated [11]. Although these recommendations were initially developed for industrial environments, they are based on universal ergonomic principles that relate working surface height to elbow position and task type. Given that surgical procedures involve precision tasks, light manipulation, and force application, these ergonomic principles are likely relevant in the operating room.

The Rapid Entire Body Assessment (REBA) is a validated and widely used tool for assessing postural load in occupational risk prevention [12]. Its application procedure and scoring criteria have been standardised by the National Institute for Safety and Health at Work (INSST) through NTP 601, which recognises its usefulness for assessing tasks that combine manipulation, force, and repetitive movements [13]. Although REBA has been used predominantly in industrial settings [12], there is growing interest in its application in healthcare [14,15,16,17] and, in particular, in the operating theatre [18]. However, no studies have quantified the effect of CCOHS working height recommendations in orthopaedic surgery using REBA.

In a previous study, our group used REBA to evaluate the optimal position of a right-handed orthopaedic surgeon performing a left total knee arthroplasty (TKA), without applying external ergonomic corrections [18]. That study identified the most favourable posture among those commonly adopted in the operating theatre, namely, facing the knee to be operated on. However, it remained unclear whether interventions such as modifying the operating table height or adjusting the surgeon’s relative working height could further reduce postural load.

The CCOHS recommendations on working heights, widely used in applied ergonomics, have not previously been evaluated in the context of orthopaedic surgery. Considering that TKA is a frequent and ergonomically demanding procedure, the objective of this study was to analyse how adjusting the working height according to CCOHS recommendations influenced the surgeon’s postural load, comparing REBA scores obtained during critical steps of the procedure with and without this ergonomic correction. We hypothesized that applying these recommendations would be associated with lower REBA scores across surgeon positions compared with standard working height.

## 2. Materials and Methods

Informed consent was obtained from the healthy volunteer who participated in the simulated surgical procedure.

### 2.1. Study Design and Simulation Model

Drawing on a previous study [18], a left TKA performed by a right-handed surgeon was selected as the model procedure for ergonomic analysis. The procedure was simulated on a healthy volunteer in an operating room environment, replicating the operative steps, positioning, and instrument handling required for TKA without involving an actual surgical case. The procedure was divided into twelve critical steps, each defined by the primary posture adopted during the corresponding surgical phase: incision (I), patellar eversion (PE), cruciate and meniscus release (CMR), tibial guide (TGC), tibial cut (TC), femoral guide (TGF), distal femoral cut (DFC) and anteroposterior cut (AFC), lavage (L), placement of definitive femoral (DFI) and tibial (DTI) implants, and closure (C).

### 2.2. Ergonomic Intervention (Working Height Correction)

**Figure 1:**
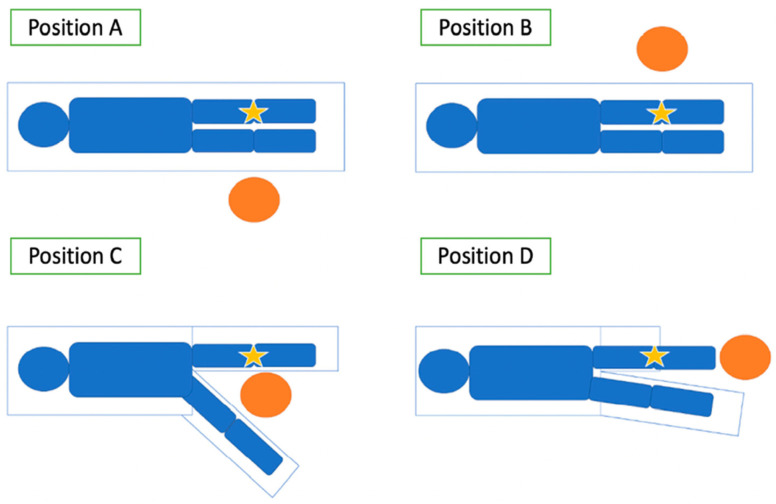
Positions for performing TKA surgery. This figure shows the different positions the surgeon can take to perform a knee replacement on the opposite side of the dominant hand. The circle represents the surgeon, and the star represents the knee to be operated on.

Postural load was analysed using the Rapid Entire Body Assessment (REBA) method. To assess the impact of working height, two surgical simulations were performed. The first simulation was performed without ergonomic adjustment, maintaining the surgeon’s usual working height (uncorrected, N), and evaluated the four positions commonly used for performing TKA: A (opposite side), B (same side), C (between the legs), and D (front), as shown in Figure 1. The second simulation used the same positions but incorporated the Canadian Centre for Occupational Health and Safety (CCOHS) recommendations for working surface height, resulting in the corrected condition (C) [11].

**Figure 2:**
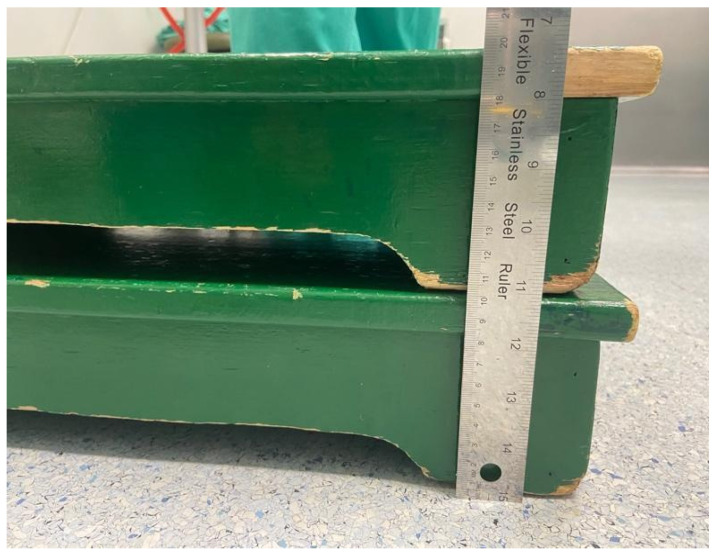
Wooden platforms (10 cm each) used to modify the surgeon’s working height during simulated surgical procedures. The height was adjusted by stacking elements of known thickness, measured in cm using the ruler shown for reference.

**Figure 3:**
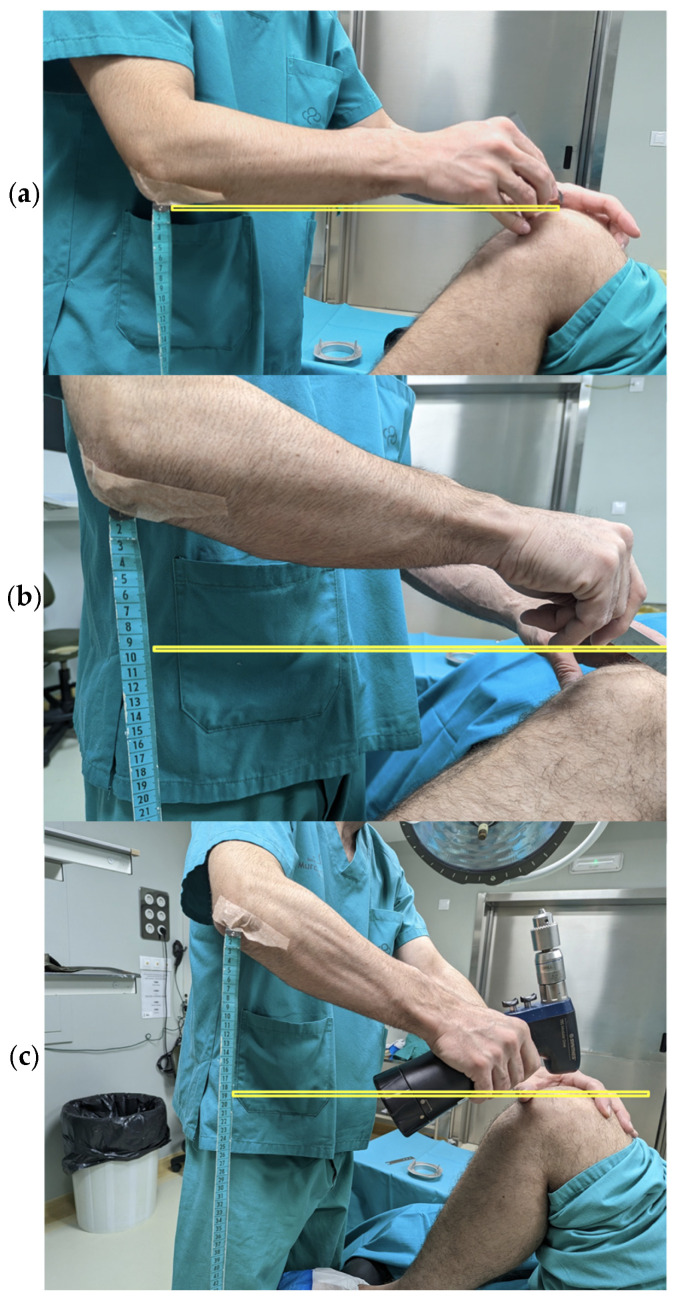
Different working heights are applied according to the surgical step. According to the recommendations of the Canadian Centre for Occupational Health and Safety (CCOHS), the optimal working height varies depending on the type of activity performed: (**a**) upper image, higher working height recommended for fine and precision tasks; (**b**) middle image, intermediate working height recommended for light tasks; and (**c**) lower image, lower working height recommended for tasks requiring the application of force.

According to CCOHS recommendations, the optimal working surface height was adjusted by task type to maintain a neutral posture and reduce musculoskeletal strain. Tasks requiring precision were performed with the working surface at elbow height or slightly above, while light tasks were performed with the working surface between five and ten centimetres below elbow level. Tasks requiring downward force were performed with the surface clearly below elbow level [10]. In the simulation, incision was classified as a precision task, lavage and closure as light tasks, and bone cutting, drilling, and prosthetic component impaction as force tasks. To adapt the working height to each task type, the surgical table and surgeon heights were adjusted using wooden risers (Figure 2), achieving the appropriate heights for each situation (Figure 3). The operating surgeon measured 178 cm in height, with a standing elbow height of approximately 108 cm. The baseline operating table height matched the surgeon’s typical working height during TKA. In the corrected condition, working height adjustments followed the CCOHS recommendations by altering the surgeon’s standing height with wooden platforms rather than adjusting the operating table. Precision tasks were conducted at elbow height, light tasks were performed 5–10 cm below elbow height, and force tasks were executed clearly below elbow level.

**Figure 4:**
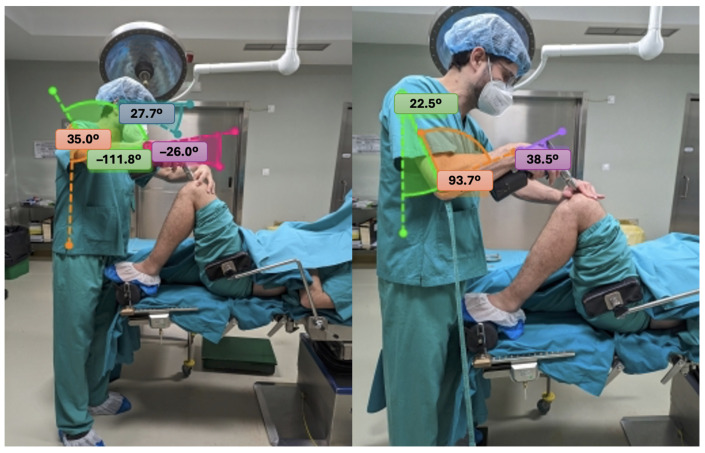
Example of angular measurements during the femoral cutting guide positioning step (FCG) in position D, without working-height correction (**left**) and with working-height correction (**right**).

Both simulations were recorded using photographs taken from less than 5 m away and at angles between 90° and 45° [19]. Images corresponding to each of the twelve critical steps, in each of the four positions, were analysed with Kinovea software (v.0.9.5) to obtain the joint angles of the neck, trunk, upper limbs, and lower limbs required for the application of the REBA method (Figure 4). Both simulations were conducted under identical conditions using standardized surgical steps. The purpose was to compare ergonomic conditions rather than evaluate surgeon performance. Although the order of simulations was fixed, the posture selection and REBA scoring were performed independently using standardized photographic frames to minimize potential bias related to learning or fatigue.

The method was applied according to the methodological guidelines previously established by our group [18], including the systematic selection of the most unfavourable representative posture at each surgical step, the application of the static posture correction factor in all steps, and the use of the repetitive action factor in steps involving the use of a surgical saw or hammer. The REBA force/load modifier was applied only in steps involving powered or impact instruments, specifically the oscillating saw and surgical hammer, which were classified as moderate-force tasks (+1) according to REBA criteria. Manual instruments were considered negligible load. Precise anatomical landmarks were used to measure joint angles. For each surgical step and surgeon position, the most unfavourable posture was defined as the frame exhibiting the greatest deviation from neutral joint alignment was visually identified. The primary evaluator selected this frame based on maximal trunk flexion, cervical inclination, shoulder elevation, or combined deviations of the upper limbs, following REBA methodological guidelines. The selected frame represented the moment during the step when the surgeon maintained the most mechanically demanding posture.

### 2.3. Postural Measurement and REBA

The study design included 12 surgical steps evaluated in 4 surgeon positions under two conditions (uncorrected (N) and corrected (C) working height), resulting in 96 distinct postures (12 × 4 × 2). For each posture, REBA scoring was performed three times: two repeated evaluations by the same observer separated by one month and one evaluation by a second observer. Consequently, a total of 288 REBA scores were obtained (Table 1).

In addition, for statistical analyses, comparisons were performed across 48 paired position–step combinations, each representing the same surgical step and surgeon position evaluated under non-corrected and corrected working-height conditions.

A summary of the number of analysed photographs, postures, and REBA evaluations is provided in Table 1 to clarify the study design and ensure consistency between the number of postures and the total REBA scores generated.

The inter- and intra-observer reliability of both the angular measurements made with Kinovea and the application of the REBA method had been previously evaluated and published by our group [18].

### 2.4. Statistical Analysis

For each surgeon position, step-specific mean REBA scores were calculated. These scores were determined by averaging three independent ratings: two sessions by rater observer 1 and one session by rater observer 2. Corrected and non-corrected conditions were compared. Paired t-tests were used to compare conditions across surgical steps (n = 12) with 95% confidence intervals. Wilcoxon signed-rank tests were conducted as sensitivity analyses. Measurement error was quantified as the minimal detectable change at 95% confidence (MC95). MC95 was calculated from the test–retest differences between the two sessions of rater observer 1 across all postures (four positions, two conditions, twelve steps). For step-wise mean scores, MC95 was obtained by dividing by the square root of three. Changes in REBA action level were assessed after rounding step-wise mean REBA scores to the nearest integer (0.5 rounded up) and applying standard REBA action thresholds. All analyses were performed using Python (version 3.13.5; Python Software Foundation, Wilmington, DE, USA) with the SciPy library, with a two-sided significance level set at alpha of 0.05.

## 3. Results

**Figure 5:**
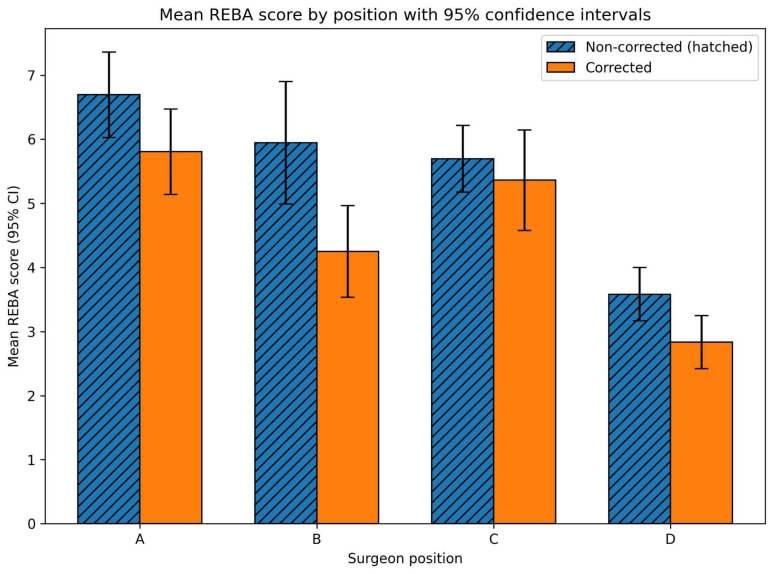
Bar chart presenting the mean global REBA scores for surgeon positions A, B, C, and D under non-corrected and corrected working-height conditions. Hatched bars denote non-corrected working height, while solid bars indicate corrected working height. Error bars represent 95% confidence intervals calculated using the t-distribution (df = 2).

The overall REBA score, calculated as the mean of the scores across the twelve surgical steps, decreased consistently when the working height was ergonomically corrected across all surgeon positions (A, B, C, and D), as illustrated in Figure 5.

These findings indicated that the ergonomic improvement associated with working-height adjustment was consistent across positions and independent of the posture. Position D, which in our previous study [18] had proven to be the most favourable from an ergonomic perspective, again showed the lowest REBA scores in both corrected and uncorrected conditions. Notably, even in this position, correcting the working height further reduced postural risk.

Mean overall REBA scores decreased from 6.69 ± 0.27 (95% CI: 6.03–7.36) (uncorrected, N) to 5.81 ± 0.27 (95% CI: 5.14–6.47) (corrected, C) in position A; from 5.94 ± 0.39 (95% CI: 4.99–6.90) (N) to 4.25 ± 0.29 (95% CI: 3.53–4.97) (C) in position B; from 5.69 ± 0.21 (95% CI: 5.17–6.22) (N) to 5.36 ± 0.32 (95% CI: 4.58–6.15) (C) in position C; and from 3.58 ± 0.17 (95% CI: 3.17–4.00) (N) to 2.83 ± 0.17 (95% CI: 2.42–3.25) (C) in position D. When expressed as whole numbers, in accordance with the urgency criteria and need for preventive action included in the REBA method [11], all scores decreased by at least one point. However, they remained within the medium-risk level (action level 2: corrective action required) for all positions except position D, where the REBA score decreased from 4 to 3, corresponding to a low-risk level with an action level of 1 (corrective action may be necessary).

**Figure 6:**
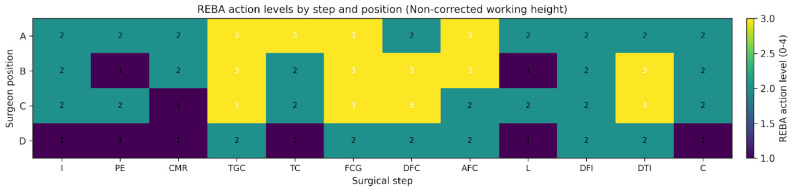
Heatmap displaying REBA action levels (0–4) by surgical step and surgeon position under non-corrected working-height conditions. Action levels are based on rounded step-wise mean REBA scores, with values of 0.5 or higher rounded up. Higher action levels indicate increased urgency for ergonomic intervention.

**Figure 7:**
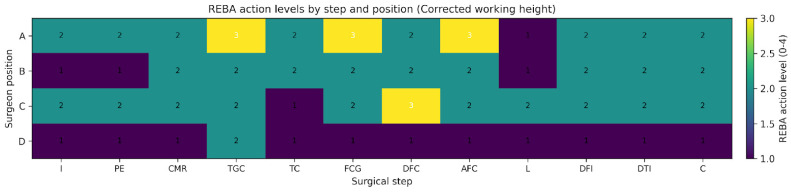
Heatmap displaying REBA action levels (0–4) by surgical step and surgeon position under corrected working-height conditions. Action levels are based on step-wise mean REBA scores rounded up to the nearest 0.5, with higher values indicating greater urgency for ergonomic intervention.

Importantly, 27 of the 48 position–step comparisons exceeded the minimal detectable change threshold, indicating that more than half of the observed reductions represented improvements beyond measurement error. Across the 12 surgical steps, working-height correction reduced mean REBA scores by 0.89 points in position A (95% CI 0.18 to 1.60; *p* = 0.019), 1.69 points in position B (95% CI 0.62 to 2.77; *p* = 0.005), and 0.75 points in position D (95% CI 0.11 to 1.39; *p* = 0.026). The change in position C was not significant (0.33 points; 95% CI −0.66 to 1.33; *p* = 0.476). When pooled across all position–step combinations (n = 48), the mean reduction was 0.92 points (95% CI 0.50 to 1.33; *p* < 0.001). The MC95, estimated from test–retest data, was 1.71 points for a single REBA rating and 0.99 points for the step-wise mean of three ratings. In total, 27 out of 48 position–step differences exceeded this threshold. Action level improved in 17 of 48 combinations, with nine transitions from action level 3 to 2 and eight from 2 to 1. Table 2 summarises inferential and interpretive metrics by position. Figure 6 and Figure 7 display action levels across steps and positions for the non-corrected and corrected conditions, respectively.

Analysis of REBA scores by surgical step showed that the highest postural load values were observed during bone-cutting, guide positioning, and prosthetic component implantation, regardless of the surgeon’s position. In all positions, correcting working height resulted in a systematic reduction in REBA scores across most surgical steps, particularly those requiring static postures maintained with trunk flexion, cervical inclination, and elevation of the dominant upper limb. Figure 6 and Figure 7 show the comparison of mean REBA scores across surgical steps for each analysed position. 

## 4. Discussion

The most important finding of the present study was that adjusting working height according to CCOHS recommendations consistently reduced surgeons’ postural load across all evaluated TKA positions, identifying working surface height as a critical and modifiable determinant of intraoperative ergonomic risk. The systematic reduction in REBA scores under corrected conditions underscores the biomechanical impact of height optimization and supports existing evidence that operating level directly affects trunk posture and upper limb loading, particularly during precision tasks and force intensive surgical steps [20,21]. Importantly, many of the observed reductions exceeded the minimal detectable change threshold, indicating that these improvements likely represent genuine postural changes rather than measurement variability. Although the numerical reductions in REBA scores appear modest, their practical significance should be interpreted within the REBA framework. In this study, more than half of the position–step comparisons exceeded the minimal detectable change established through reliability analysis, indicating that these reductions are unlikely to be attributable to measurement error. Additionally, several transitions to lower REBA action levels were observed, reflecting reduced ergonomic risk and a diminished need for corrective intervention. Specifically, 27 of the 48 paired position–step comparisons exceeded the minimal detectable change threshold, supporting the interpretation that the observed improvements reflect genuine postural changes rather than measurement variability.

Although CCOHS recommendations originate from industrial ergonomics, the underlying concept of adapting working height to task type is grounded in human biomechanics and is therefore transferable to surgical environments where comparable manual tasks are performed. Moreover, several ergonomic assessment methods currently used in healthcare, including the Rapid Entire Body Assessment (REBA) method applied in this study, were also originally developed in industrial contexts and later adopted for the evaluation of musculoskeletal risk in clinical environments.

The high prevalence of musculoskeletal disorders among orthopaedic surgeons reported in the literature reinforces the clinical relevance of these findings. Multiple systematic reviews and observational studies have shown that more than 60–70% of orthopaedic surgeons experience pain or functional limitations related to their professional activities [1,2,3]. This issue extends beyond orthopaedics, affecting healthcare professionals who are exposed to sustained postures and physically demanding tasks [22,23,24]. The magnitude of this problem highlights the necessity for effective preventive interventions to reduce sustained postural load during surgical practice.

Several intraoperative factors have been identified as contributors to increased musculoskeletal risk, including procedure duration, surgical technique, and the use of specific equipment [25,26,27]. The present results are consistent with this evidence and offer a practical strategy, demonstrating that adjusting working height can reduce ergonomic risk regardless of the surgeon’s position. This reduction was most pronounced during surgical phases requiring caudal force, such as bone cutting, drilling, and prosthetic component impaction, during which ergonomic correction reduced trunk flexion and abduction of the dominant shoulder. This pattern aligns with studies reporting high physical load associated with open surgery and procedures requiring sustained manual force [25,26,27].

Application of the REBA method enabled quantitative evaluation of ergonomic risk and facilitated comparison with the existing literature. Postural analysis tools such as REBA have demonstrated utility in identifying musculoskeletal risk in healthcare settings, including among professionals exposed to sustained postures and precision movements, such as dentists, physical therapists, ophthalmology residents, and those performing endoscopic techniques [13,14,15,16]. In these groups, REBA has identified high-risk postures during prolonged surgical procedures that, similar to orthopaedic surgery, involve sustained cervical flexion, trunk tilt, and elevation of the dominant upper limb. This comparability in postural patterns supports the appropriateness of REBA for assessing ergonomic risk in surgical procedures, despite differences in external load between specialties.

Additionally, these results complement previous findings by this research group, which identified the position in front of the operated knee, at the patient’s feet, as the posture associated with the lowest ergonomic risk for performing contralateral TKA relative to the dominant hand [18].

From a training perspective, a significant gap exists in ergonomic education during surgical training. Studies across various contexts indicate that most residency programmes lack formal ergonomic training, despite the high prevalence of musculoskeletal disorders among surgeons in training [28,29,30,31,32]. This deficiency has been documented in American, European, and Asian training programmes and contrasts with evidence supporting the benefits of educational interventions. Structured prevention and ergonomic training programmes have demonstrated significant improvements in knowledge, postural awareness, and reductions in musculoskeletal symptoms among residents and practising surgeons [32,33,34].

The present findings support the perspective that ergonomics should be regarded as a structural component integrated into surgical planning rather than a one-time adjustment. This approach aligns with recommendations to incorporate ergonomic principles into the organisation of the surgical environment and the design of care protocols [9,35,36]. Furthermore, emerging technologies provide opportunities for objective assessment of postural load. Specifically, portable ergonomic monitoring devices have demonstrated increasing potential for integration into assessment and training programmes in healthcare settings [37].

This study presents several limitations, including the simulation’s static nature and the analysis of a single surgeon performing a single surgical procedure.

The simulation involved a single right-handed surgeon performing a standardized procedure. While this design allowed isolation of the ergonomic effect of working height, it may limit generalizability to surgeons with varying anthropometric characteristics, handedness, operative habits, or operating room layouts. Additionally, differences in operating room configuration, imaging equipment, or surgical team dynamics may affect intraoperative posture. Furthermore, it would not be feasible to reproduce the same surgical procedure twice in vivo in the same patient to compare corrected and non-corrected working heights under identical conditions. Performing the procedure on different patients would introduce additional variability related to anatomy, body mass index, and operative conditions. For this reason, a simulation-based design was considered appropriate to allow controlled ergonomic comparisons.

The postural analysis was based on two-dimensional photographic assessment rather than dynamic three-dimensional motion capture, and no direct physiological measurements such as electromyography or muscle fatigue assessment were performed. Although this approach is widely used in ergonomic research and validated for angular measurement [19], it may introduce minor measurement errors related to camera perspective or limb rotation.

The Rapid Entire Body Assessment (REBA) method provides an estimate of postural ergonomic risk but does not directly quantify internal biomechanical load. In addition, the simulated procedure was conducted on a healthy volunteer with normal body habitus. Patient morphology, especially elevated body mass index, can affect surgical exposure and working height in actual procedures. Furthermore, intraoperative variables such as tissue resistance, surgical assistance, and dynamic movements during live surgery may affect the surgeon’s posture. These variables were deliberately controlled in the current simulation to enable standardized ergonomic assessment; however, they should be considered when interpreting the results. Controlling these variables in a simulated setting allowed the study to isolate the ergonomic effect of working-height correction while minimizing confounding factors related to patient anatomy and intraoperative variability.

In addition, complete rater blinding was not feasible because the working-height correction could be visually identified in the photographic images. However, the use of repeated scoring sessions and two independent evaluators was intended to reduce potential scoring bias.

Finally, the simulated model did not replicate the full biomechanical resistance observed in live surgery, including bone-cutting resistance, drilling forces, and soft-tissue tension. Consequently, the force component in REBA scoring was estimated based on the type of surgical task rather than measured directly. While this method enabled standardized comparisons between conditions, future studies conducted during live surgical procedures are necessary to further validate these findings [25,32,38].

Despite these limitations, this study provides evidence with direct clinical implications. Modern operating tables permit precise height adjustments, and clear ergonomic recommendations are available; however, their systematic implementation in clinical practice remains limited [6,18]. The present findings support the integration of evidence-based ergonomic recommendations into institutional protocols and targeted programmes. Recent systematic reviews have confirmed the effectiveness of structured ergonomic interventions in reducing musculoskeletal disorders among surgeons [36].

Collectively, these results demonstrate that working surface height is a key determinant of postural load during TKA and that adjustment in accordance with CCOHS recommendations can significantly reduce the surgeon’s musculoskeletal risk. These findings reinforce the importance of considering incorporating ergonomics into routine surgical practice, thereby contributing to safer and more sustainable professional practice.

## 5. Conclusions

Adjusting working height according to CCOHS recommendations reduced surgeons’ postural load during simulated TKA across all evaluated operating positions, as demonstrated by consistently lower mean REBA scores under corrected conditions. The most substantial reductions were observed during force-intensive surgical steps. Following ergonomic correction, the position in front of the operated knee at the patient’s feet achieved a low-risk action level. These findings suggest that evidence-based working-height adjustment is a practical and effective ergonomic intervention and supports considering its integration into routine orthopaedic surgical practice. Future controlled studies with larger surgeon samples are warranted to confirm these findings.

## Figures and Tables

**Table 1:** Study design and REBA workflow. A total of 96 photographs (48 per condition: non-corrected [N] and corrected [C]) were analyzed, generating 96 postures (12 steps × 4 positions × 2 conditions). Each posture was rated three times, resulting in 288 REBA scores.

Study Component	Description	Number
Photographs per condition	One photograph for each surgical step and surgeon position	48 per condition
Conditions	Non-corrected (N) and corrected (C) working height	2
Total photographs analysed	48 × 2 conditions	96 photographs
Unique postures	12 steps × 4 positions × 2 conditions	96 postures
Ratings per posture	Observer 1 (session 1), Observer 1 (session 2), Observer 2 (session 1)	3 ratings
Total REBA scores generated	96 postures × 3 ratings	288 REBA scores

**Table 2:** Corrected versus non-corrected differences by surgeon position. Mean difference is expressed as non-corrected minus corrected (N-C) across 12 steps. MC95 threshold is 0.99 points for the step-wise mean of three ratings.

Position	Mean Difference (N-C), Points (95% CI)	*p*	|Δ| > MC95 (n/12)	Action Level Improved, n/12
A	0.89 (0.18 to 1.60)	0.019	9/12	2/12
B	1.69 (0.62 to 2.77)	0.005	8/12	6/12
C	0.33 (−0.66 to 1.33)	0.476	5/12	4/12
D	0.75 (0.11 to 1.39)	0.026	5/12	5/12

## Data Availability

Data are available from the corresponding author upon reasonable request.

## Abbreviations

CCOHS, Canadian Centre for Occupational Health and Safety; DFC, distal femoral cut; DFI, definitive femoral implant; DTI, definitive tibial implant; INSST, National Institute for Safety and Health at Work; REBA, Rapid Entire Body Assessment; TKA, total knee arthroplasty; WMSDs, work-related musculoskeletal disorders.

## References

[1] Vasireddi N.M., Vasireddi N.B., Shah A.K.B., Moyal A.J., Gausden E.B., Mclawhorn A.S.M., Poelstra K.A., Gould H.P., Voos J.E., Calcei J.G. (2024). High prevalence of work-related musculoskeletal disorders and limited evidence-based ergonomics in orthopaedic surgery: A systematic review. Clin. Orthop. Relat. Res..

[2] Epstein S., Sparer E.H., Tran B.N., Ruan Q.Z., Dennerlein J.T., Singhal D., Lee B.T. (2018). Prevalence of work-related musculoskeletal disorders among surgeons and interventionalists: A systematic review and meta-analysis. JAMA Surg..

[3] Buddle V., Nugent R., Jack R.A., DeLuca P. (2023). Orthopedists report high prevalence of work-related pain and low ergonomic awareness. Orthopedics.

[4] Valtanen R.S., van Niekerk M., Chu C.R. (2025). Ergonomics in the operating room: Recommendations for orthopaedic surgeons. J. Am. Acad. Orthop. Surg..

[5] Moldovan F., Moldovan L. (2023). Fair healthcare practices in orthopedics assessed with a new framework. Healthcare.

[6] Aaron K.A., Vaughan J., Gupta R., Ali N.-E., Beth A.H., Moore J.M., Ma Y., Ahmad I., Jackler R.K., Vaisbuch Y. (2021). The risk of ergonomic injury across surgical specialties. PLoS ONE.

[7] Alostaz M., Bansal A., Gyawali P., Louie P.K. (2024). Ergonomics in spine surgery: A systematic review. Spine.

[8] Haddad A., Lendoire M., Ito K., Ayabe R.I., Maki H., Pietz J.T., Stucky C.-C.H., Tzeng C.-W.D., Cao H.S.T., Chun Y.S. (2025). Ergonomic considerations in open liver surgery. J. Gastrointest. Surg..

[9] Barrios E.L., Polcz V.E., Hensley S.E., Sarosi G.A., Mohr A.M., Loftus T.J., Upchurch G.R., Sumfest J.M., Efron P.A., Dunleavy K. (2023). Ergonomic problems, principles and potential solutions in surgical operations. Surgery.

[10] Mah A.B., Alam F.M.M., Larouche J.M.M., Dandal M.-A., Cohen T., Hallbeck S., Norasi H., Kallocsai C., Sriram S.D.M., Helman J.D. (2024). Operating room ergonomics needs and priorities: A survey of operating room staff. Ann. Surg..

[11] Canadian Centre for Occupational Health and Safety Working in a Standing Position—Basic Information. CCOHS. https://www.ccohs.ca/oshanswers/ergonomics/standing/standing_basic.html.

[12] Hita-Gutiérrez M., Gómez-Galán M., Díaz-Pérez M., Callejón-Ferre Á.J. (2020). An overview of REBA method applications in the world. Int. J. Environ. Res. Public Health.

[13] Instituto Nacional de Seguridad y Salud en el Trabajo (2003). NTP 601: Evaluación de las Condiciones de Trabajo. Método REBA.

[14] Căteanu A.M.L., Repanovici A., Baritz M.I., Scutariu M.M., Ostafe A.I.T., Pantea I. (2024). Postural risks in dental practice: An assessment of musculoskeletal health. Sensors.

[15] Tasso M., Menoni O. (2025). REBA integrated with organisational analysis to assess the risk of biomechanical overload in physiotherapists. Ergonomics.

[16] Morrison A.K., Kumar S., Amin A., Urban M., Kleinman B. (2024). An ergonomic risk assessment of ophthalmology residents using the Rapid Entire Body Assessment (REBA) scale. Cureus.

[17] Pawa S., Kwon R.S., Fishman D.S., Thosani N.C., Shergill A., Grover S.C., Al-Haddad M., Amateau S.K., Buxbaum J.L., Calderwood A.H. (2023). ASGE guideline on the role of ergonomics for prevention of endoscopy-related injury: Methodology and review of evidence. Gastrointest. Endosc..

[18] Sánchez-Robles M., Díaz-Martínez F.J., León-Muñoz V.J., Marín-Martínez C., Murcia-Asensio A., Moreno-Cascales M., Lajara-Marco F. (2023). Ergonomic evaluation of different surgeon positions for total knee arthroplasty surgery. Appl. Sci..

[19] Puig-Diví A., Escalona-Marfil C., Padullés-Riu J.M., Busquets A., Padullés-Chando X., Marcos-Ruiz D. (2019). Validity and reliability of the Kinovea program in obtaining angles and distances using coordinates in four perspectives. PLoS ONE.

[20] van Veelen M.A., Jakimowicz J.J., Kazemier G. (2002). Assessment of the ergonomically optimal operating surface height for laparoscopic surgery. Surg. Endosc..

[21] Manasnayakorn S., Cuschieri A., Hanna G.B. (2009). Ergonomic assessment of optimum operating table height for hand-assisted laparoscopic surgery. Surg. Endosc..

[22] Jacquier-Bret J., Gorce P. (2023). Prevalence of body area work-related musculoskeletal disorders among healthcare professionals: A systematic review. Int. J. Environ. Res. Public Health.

[23] Kriseman M.L., Wang W.-L., Sullinger J., Schmeler K.M., Ramirez P.T., Herzog C.E., Frumovitz M. (2012). Physical strain and need for ergonomic training among gynecologic oncologists. Gynecol. Oncol..

[24] Arden D., Seifert E., Donnellan N., Guido R., Lee T., Mansuria S. (2013). Ergonomic deficits in robotic gynecologic oncology surgery. J. Minim. Invasive Gynecol..

[25] Yang L., Money S.R., Morrow M.M., Lowndes B.R., Weidner T.K., Fortune E., Davila V.J., Meltzer A.J., Stone W.M., Hallbeck S.M. (2020). Impact of procedure type, case duration, and adjunctive equipment on surgeon intraoperative musculoskeletal discomfort. J. Am. Coll. Surg..

[26] Yang L., Wang T., Weidner T.K., Madura J.A., Morrow M.M., Hallbeck M.S. (2021). Intraoperative musculoskeletal discomfort and risk for surgeons during open and laparoscopic surgery. Surg. Endosc..

[27] Fan X., Forsman M., Yang L., Lind C.M., Kjellman M. (2022). Surgeons’ physical workload in open surgery versus robot-assisted surgery and non-surgical tasks. Surg. Endosc..

[28] Walsh C.M., Qayed E., Aihara H., Anand G.S., Byrne K., Chahal P., Dacha S., James T.W., Kowalski T.E., Repaka A. (2021). Core curriculum for ergonomics in endoscopy. Endoscopy.

[29] Epstein S., Tran B.N., Capone A.C., Ruan Q.Z., Fukudome E.Y., Ricci J.A., Testa M.A., Dennerlein J.T., Lee B.T.M., Singhal D. (2019). The current state of surgical ergonomics education in U.S. surgical training: A survey. Ann. Surg..

[30] Jensen M.J., Liao J., Van Gorp B., Sugg S.L., Shelton J., Corwin C., Lal G. (2021). Incorporating surgical ergonomics education into surgical residency curriculum. J. Surg. Educ..

[31] Shah M., Gross K., Wang C., Kurlansky P., Krishnamoorthy S. (2024). Working Through the Pain: A Cross-Sectional Survey on Musculoskeletal Pain Among Surgeons and Residents. J. Surg. Res..

[32] Cerier E., Hu A., Goldring A., Rho M., Kulkarni S.A. (2022). Ergonomics workshop improves musculoskeletal symptoms in general surgery residents. J. Surg. Res..

[33] Giagio S., Volpe G., Pillastrini P., Gasparre G., Frizziero A., Squizzato F. (2019). A preventive program for work-related musculoskeletal disorders among surgeons: Outcomes of a randomized controlled clinical trial. Ann. Surg..

[34] Hess P., Athanasiadis D., Lee N.K., Monfared S., Cleveland P.M., Stefanidis D. (2024). Preventing surgeon work-related musculoskeletal disorders: A pilot study of the comprehensive operating room ergonomics (CORE) program. Am. J. Occup. Ther..

[35] Restaino S., D’indinosante M., Perelli F., Arcieri M., Cherchi V., Petrillo M., Cavaliere A.F., Cianci S., Pellecchia G., Meniconi R.L. (2024). Ergonomics in the operating room and surgical training: A survey on the Italian scenario. Front. Public Health.

[36] Choi S.D. (2012). A review of the ergonomic issues in the laparoscopic operating room. J. Med. Eng. Technol..

[37] Sabino I., Fernandes M.D.C., Cepeda C., Quaresma C., Gamboa H., Nunes I.L., Gabriel A.T. (2024). Application of wearable technology for ergonomic risk assessment of healthcare professionals: A systematic review. Int. J. Ind. Ergon..

[38] Santos W., Rojas C., Isidoro R., Lorente A., Dias A., Mariscal G., Benlloch M., Lorente R. (2025). Efficacy of ergonomic interventions on work-related musculoskeletal disorders: A systematic review and meta-analysis. J. Clin. Med..

